# The role of cow urine in the oviposition site preference of culicine and *Anopheles *mosquitoes

**DOI:** 10.1186/1756-3305-4-184

**Published:** 2011-09-26

**Authors:** Eliningaya J Kweka, Eunice A Owino, Beda J Mwang'onde, Aneth M Mahande, Mramba Nyindo, Franklin Mosha

**Affiliations:** 1 Tropical Pesticides Research Institute, Division of Livestock and Human disease vectors control, Mosquitoes Section, P. O. Box 3024, Arusha, Tanzania; 2 Kilimanjaro Christian Medical College of Tumaini University, P. O. Box 2240, Moshi Tanzania; 3 School of Biological Sciences, University of Nairobi, P. O. Box 30197 - 00100, Nairobi, Kenya

## Abstract

**Background:**

Chemical and behavioural ecology of mosquitoes plays an important role in the development of chemical cue based vector control. To date, studies available have focused on evaluating mosquito attractants and repellents of synthetic and human origins. This study, however, was aimed at seasonal evaluation of the efficiency of cow urine in producing oviposition cues to *Anopheles gambiae *s.l. and *Culex quinquefasciatus *in both laboratory and field conditions.

**Methods:**

Oviposition response evaluation in laboratory conditions was carried out in mosquito rearing cages. The oviposition substrates were located in parallel or in diagonal positions inside the cage. Urine evaluation against gravid females of *An. arabiensis *and *Cx. quinquefasciatus *was carried out at Day 1, Day 3 and Day 7. Five millilitres (mls) of cow urine was added to oviposition substrate while de-chlorinated water was used as a control. In field experiments, 500 mls of cow urine was added in artificial habitats with 2500 mls of de-chlorinated water and 2 kgs of soil. The experiment was monitored for thirty consecutive days, eggs were collected daily from the habitats at 7.00 hrs. Data analysis was performed using parametric and non-parametric tests for treatments and controls while attraction of the oviposition substrate in each species was presented using Oviposition Activity Index (OAI).

**Results:**

The OAI was positive with ageing of cattle urine in culicine species in both laboratory and field experiments. The OAI for anopheline species was positive with fresh urine. The OAI during the rainy season was positive for all species tested while in the dry season the OAI for culicine spp and *Anopheles gambiae *s.l., changed with time from positive to negative values.

Based on linear model analysis, seasons and treatments had a significant effect on the number of eggs laid in habitats, even though the number of days had no effect.

**Conclusion:**

Oviposition substrates treated with cow urine in both laboratory and field conditions have shown that cow urine left to age from 1-7 days has an influence on oviposition behavioural response in mosquitoes. The analysis of microbial colonies for decaying urine should be investigated along with its associated by-products.

## Background

Oviposition site selection by gravid mosquitoes is one of the crucial events in non-parental organisms [1-6]. In mosquitoes, the reproductive success depends on the ability of a mosquito to search for the suitable oviposition site that will ensure survival for the progeny [7,8]. Habitat related chemical cues have been shown to play an important role in the oviposition site selection by gravid female mosquitoes [9,10]. *Anopheles gambiae *s.s. and *Culex quinquefasciatus *mosquitoes are anthropophilic, thus, highly associated with habitats close to human dwellings [7,11,12], but *An. arabiensis *mosquitoes in particular are anthropophilic or zoophilic depending on geographical location and host availability [13,14]. In different areas, *Cx. quinquefasciatus *have been found laying egg rafts in habitats with higher levels of organic pollutants [15,16], while *An. gambiae *s.s. and *An. arabiensis *have been found to lay eggs in turbid small water pools with short vegetation or without vegetation [17,18]. Micro-organisms inhabiting mosquito breeding sites have been found to play a major role in the decomposition of detritus and other nutrients present in these habitats, leading to the production of several metabolites emanating different chemical cues [19]. Gravid mosquitoes are often attracted to water in spite of the fact that the decision to lay eggs may depend on additional olfactory signals or other stimuli received when the insect lands on the water surface [5,20-22]. Therefore, interruption of oviposition site selection for mosquitoes may lead to population growth reduction and have a positive impact in control programmes.

Using resting boxes baited with cow urine [23,24] and cow as bait in an odour-baited trap, high densities of female *An. gambiae *s.l. were sampled [25,26]. It is further known that most of the sampled females were either semi-gravid or fully gravid [23,24]. These results gave rise to the hypothesis that, cow urine-polluted habitats influence the attraction of gravid female mosquitoes for oviposition. Other arthropods have been shown to be attracted to the contents of cow urine i.e., (4-methylphenol, 3-n-propylphenol) during host seeking experiments [27]. Sampling of *Hybomitra *horseflies increased by 1.5-1.9 times when cow urine was used as bait in the traps [27]. To build up on the foundation that has been laid by these observations, it would be necessary to set up experiments that assess the responses of gravid mosquitoes to artificial habitats baited with different stages of ageing cow urine.

Therefore, the aim of this study was to assess the oviposition response of *An. gambiae *s.l. and *Cx. quinquefasciatus *mosquitoes in laboratory and field conditions baited with cow urine.

## Materials and methods

### Laboratory experiments

Laboratory experiments were carried out at the Tropical Pesticides Research Institute field station, located at lower Moshi rice irrigation schemes (37°20' E, 3°21' S; 750 M above sea level). *An. arabiensis *and *Cx. quinquefasciatus *mosquitoes (Mabogini strain, colonised in insectaries since 2006) were used. Mosquitoes were blood fed using shaved restrained rabbits at the age of 3 days after emergence. Both *An. arabiensis *and *Cx. quinquefasciatus *were allowed to feed on the rabbit for duration of one hour in the laboratory. Both species were monitored for 48 to 96 hrs post blood meal until they were fully gravid. The insectary temperature was maintained at 27.4 ± 1°C with a relative humidity of 70 to 80%. The light regime in the insectary was 12L:12D [28]. Gravid females of both *An. arabiensis *and *Cx. quinquefasciatus *were provided with sugar solution (10% sucrose) during oviposition experiments.

### Oviposition choices

One fully gravid female mosquito of *An. arabiensis *or *Cx. quinquefasciatus *(48^th ^to 96^th ^hr after full blood meal) was placed in a cage (30 × 30 × 30 cm), made from iron rods and covered with fine netting material. Experiments were set up using the oviposition requirements and substrates specific for each species (i.e. a wet cotton wool covered by filter paper on a Petri dish for *An. arabiensis *and a three quarters water filled plastic cup for *Cx. quinquefasciatus*). The composition of the solution used in oviposition substrate was in the ratio of 1:5 for cow urine and water respectively. Each experiment used 1, 3 and 7 day old cow urine and had twenty replicates for each species. A total of 60 gravid female mosquitoes of each species were used for evaluations with the three ageing urine sets. Oviposition choice experiments were made either in parallel or diagonal arrangements. In parallel oviposition choice experiments, the oviposition substrates were placed 20 cm apart in the centre of the cage. In diagonal oviposition experiments, the choice experiment was set diagonally (two oviposition substrates were placed in cage corners diagonally to each other).

In both settings the treatments were made up using urine collected from a 2 year old and zero grazed female Zebu cow to avoid variations [23,29]. The urine was collected between 6 to 7 am daily by the cow owner in a plastic basin. On day one, fresh urine was used directly in laboratory experiments in a ratio of 1:5. Urine for experiments on days 3 and 7 was aged in open plastic basins to allow microbial decomposition. The oviposition substrates were wetted with three treatments of either 5 mls of fresh urine (Day 1), three day old (stored for 3 days) or seven day old urine (stored for 7 days) diluted in 25 mls of de-chlorinated water. De-chlorinated water was used as a control in all experiments. Each experiment had ten replicates; the numbers of eggs laid were counted at 7:30 am the next day. The protocol for laboratory experiments was adapted from Sumba *et al. *[22] with some modifications, under the assumption that mosquitoes lay their eggs randomly.

### Design of Field experiments

Field experiments were carried out outside human dwellings at lower Moshi rice irrigation schemes, located 19 km south of Moshi town, on the lower slopes of Mount Kilimanjaro. The study area is described in detail elsewhere by Kweka *et al. *[23,29]. The experiments were carried out in two seasons: during the dry season (January to February) and the rainy season (mid March to May). The treatment and the control were placed five metres apart. A pair comprising of a treatment and control were placed ten metres apart from each other.

The experimental set up design was the same for both the dry and wet season, since oviposition site selection for *Aedes aegypti *is more affected by seasons [30,31]. Munga *et al., *[32] found that in the dry season, *An. gambiae *significantly deposited more eggs in water from farmlands compared to water from forests and natural wetlands. However, the differences were statistically insignificant during the wet season. Furthermore, substrate composition and concentration have been found to influence the oviposition site selection for *An. gambiae *[5]. *An. gambiae *s.l. eggs were collected carefully using a magnifying hand lens to ensure accuracy.

### Dry season oviposition choice in small scale field experiments

About twenty artificial habitats were created by mixing 2 kilograms of soil, 2500 mls of chlorine free water and 500 mls of cow urine (a similar ratio of 1:5 for cow urine and water was used as in the laboratory experiments). These were placed in washing basins measuring 35 cm in diameter and 14 cm in depth. The inner side of each basin was lined up with white paper. The control habitats were made with the same composition of resources but excluded the cow urine. Mosquito eggs were counted daily for thirty days. Water with respective aged urine levels in habitats was monitored and when decreased/evaporated was replenished with similar ratios.

### Rainy season oviposition choice in field experiments

Experimental and artificial habitats were set up, egg laying and monitoring schedules were similar to the ones used in the dry season.

### Data Analysis

In laboratory experiments, data were recorded at 7:30 hrs while in field experiments set up was daily every morning at 7:30 hrs. Due to high variations in the number of eggs laid in each treatment and between replicates, the number of eggs laid was log (n + 1) transformed. Following log transformation, the normally distributed data were analysed using the parametric test while a non-parametric test was used for data that were not normally distributed. For field data, the comparison between treatments and controls were carried out using the Kruskal Wallis test. The comparisons of different factors associated with oviposition choice of gravid mosquitoes in different habitats were analysed using the general linear model multivariate analysis (MANOVA). Number of eggs laid was used as the dependent variable, while seasons, treatments and species were used as covariates and days were used as random factor. Data analysis was performed using PASW statistics version 18 (SPSS Inc., Chicago, IL).

The oviposition attraction of cow urine was scored by oviposition activity index (OAI), which was calculated using the protocol used by Hwang *et al. *[33]. OAI = (N_T_-N_S_)/(N_T _+ N_S_) where N_T _denotes the mean number of eggs laid in the treated substrate and N_S _denotes the mean number of eggs laid in the control substrate. All the index values ranged from -1 to +1. Positive OAI means that more eggs were laid in the treatment substrate than in control while, for the negative OAI, more eggs were laid in the control than in treatment substrate.

## Results

### Laboratory experiments

For the diagonal oviposition choice setup, the mean number of eggs laid in oviposition substrates treated with fresh urine (1 day) was significantly higher compared to eggs laid in the control substrates, for *An. arabiensis *(t = 12.71, d.f. = 1, *P *= 0.010) and *Cx. quinquefasciatus *(t = 12.71, d.f. = 1, *P *= 0.030). For the oviposition substrates treated with 3 day old cow urine, more eggs were laid in the control than in treatment substrates, for *An. arabiensis *(t = 12.71, d.f. = 1, *P *= 0.010) while for *Cx. quinquefasciatus*, more eggs were laid in the treatment than in control substrates (t = 12.71, d.f. = 1, *P *= 0.050). For oviposition substrates treated with 7 day old cow urine, the number of *An. arabiensis *eggs laid were significantly higher in control than in treatment substrates (t = 12.71, d.f = 1, P = 0.030); for *Cx. quinquefasciatus*, the number of eggs laid in treatment substrates were significantly higher than those in the control substrates (t = 12.71, d.f = 1, P = 0.030).

In parallel choice experiments, the number of eggs laid in fresh urine treated substrates by *An. arabiensis *was significantly higher than in control substrates (t = 12.71, d.f. = 1, *P *= 0.010). In *Cx. quinquefasciatus *eggs laid in treatments and control were statistically similar (t = 12.71, d.f. = 1, *P *= 0.350). In results from the 3 day old cow urine in the parallel set up, *An. arabiensis *laid more eggs in the control than in treatment group (t = 12.71, d.f. = 1, *P *= 0.020) and *Cx. quinquefasciatus *laid more eggs in the treatments than in the control group (t = 12.71, d.f. = 1, *P *= 0.010). In 7 day old cow urine in parallel oviposition choice, *An. arabiensis *significantly oviposited more eggs in the control than in the treatment groups (t = 12.71, d.f. = 1, *P *= 0.010) while *Cx. quinquefasciatus *significantly oviposited more eggs in the treatment substrates than in control substrates (t = 12.71, d.f. = 1, *P *= 0.010).

OAI for both *An. arabiensis *and *Cx. quinquefasciatus *ranged from -1 to + 1 in treatments throughout all experimental days. In one day-old urine, both diagonal and parallel experimental settings recorded more eggs than in the control groups for both species (Table 1). For the 3 day old urine, *An. arabiensis *laid more eggs in the control for both parallel and diagonal settings resulting in a negative OAI. However, *Cx. quinquefasciatus *laid more eggs in treatments that resulted in positive OAI values (Table 1). For the 7 day old urine, *An. arabiensis *treatment replicates in both the parallel and diagonal settings recorded fewer eggs than in the control groups, which resulted in negative OAI while *Cx. quinquefasciatus *recorded more eggs in treatment replicates resulting in a positive OAI value (Table 1).

**Table 1:** Oviposition activity index of mosquito species during the insectary experiments in different substrates and settings.

	Fresh urine (immediately collected		3 days old		7 days old	
	**Diagonal**	**Parallel**	**Diagonal**	**Parallel**	**Diagonal**	**Parallel**

*An. arabiensis*	0.93	0.70	-0.90	-0.99	-1.00	-1.00

*Cx. quinquefasciatus*	0.92	0.39	0.98	1.00	1.00	1.00

### Field experiments

In the dry season, the thirty days records for *An. gambiae *s.l. eggs in treatment replicates were significantly higher than those from controls (t = 2.26, d.f. = 29, *P <*0.010); a similar trend was observed for culicine spp (t = 2.26, d.f. = 29, *P *< 0.010). Nevertheless, the number of eggs of *An. gambiae *s.l. decreased with the increasing number of days while for culicine spp, increased with days.

In the rainy season, the number of eggs laid in urine treated oviposition substrates were statistically similar from control substrates for both *An. gambiae *s.l. (t = 2.26, d.f. = 29, *P *= 0.790) and culicine spp (t = 2.26, d.f. = 29, P = 0.790).

General linear model analysis revealed that treatments, species, and seasons significantly influenced oviposition; days had no influence on oviposition substrates selection by gravid mosquitoes (Table 2).

**Table 2:** Analysis of the factors associated with the influence of gravid mosquitoes oviposition in artificial habitats for thirty days of field evaluations.

Source of variation	F-test	P-values
Season	16 _1, 238_	<0.001

Days	0.32 _29, 210_	0.99

Treatments	7.07 _1, 238_	<0.01

Species	19.4 _1, 238_	<0.001

* (The source of variation was considered significant at *P <*0.05).

The OAI were found to shift from positive to negative values and vice-versa. The OAI values shifted from negative (-0.25) to positive (0.25) for culicine spp and changed from positive (0.48) to negative (-0.28) for *An. gambiae *s.l. throughout the study and from Day 1 to Day 30, respectively.

## Discussion

The results of this study suggest that *An. gambiae *s.l. are attracted to ovipositing in habitats with fewer organic pollutants in both laboratory and field conditions while culicines have been shown to be attracted to oviposit in habitats with decomposed organic contents. These findings are consistent with our hypothesis that in culicine mosquito species oviposition cues come from the decomposed organic substrates while *An. gambiae *s.l. respond to cues from habitats without decomposing matter. This response was clearly observed in field experiments, which resulted in an oviposition shift between *An. gambiae *s.l. and culicine spp. Laboratory findings showed that experimental design had no influence on oviposition. Yet, treatments were found to influence oviposition. The microbial activities on urine decomposition have been assumed to produce different cues and hence attract mosquito species differently as bacterial colonies in habitats have an influence on chemical cues released from these habitats. Different chemical cues have been found to influence species for oviposition in different natural habitats [7,8,34,35].

In previous studies, it was observed that the oviposition of *An. arabiensis *in rice fields increased with first application of organic fertilizer while subsequent applications increased *Cx. quinquefasciatus *mosquitoe*s *[21,36-38]. Additions of manure and environmental manipulation in farms have been shown to decrease the population of anopheline species and increase the population of culicine species [36,37,39].

Four compounds have been identified from fresh cow urine; these are 3- and 4-methyl phenol, 3-ethylphenol, 3-n-propylphenol and 2-methoxyphenol [40,41]. These compounds have electro-antennographical effects on mosquitoes and *Glossina spp *[40,41]. The attractant and/or repellent effect of cow urine to *An. arabiensis *and *Cx. quinquefasciatus *in our experiments could be due to the fact that the four chemical compounds formerly identified from cow urine were active. Due to microbial activities, the chemical compounds could have been produced as by-products that might influence oviposition attraction and/or deterrence cues for each mosquito species. Furthermore, the presence of chemicals and continued decomposition of cow urine increases microbial colonies, which might have influenced the observed results. The increase in microbial colonies in habitats generates more volatile compounds that attract gravid mosquitoes' to oviposit [5,7,21,38].

Addition of cow urine to habitats attracted oviposition by anopheline species in the early day intervals while in the later ones culicines were attracted more [16,36,37,42]. The trend in decreased oviposition observed in *An. gambiae *s.l. during the dry season might have been due to the hot weather which, increased evaporation rate of water and hence increased the concentration of the contents in the habitats and subsequently the microbial activity outputs. Consequently, in the rainy season, oviposition was observed to be low due to the effect of frequent dilution of habitat substrate by rain and the presence of alternative natural habitats. This variation caused a change in the number of eggs laid by each species per habitat and treatment. Oviposition studies previously carried out in Tanzania showed that, the residue of skatole and synthetic oviposition pheromone in the field against culicine spp were strong for one week (7 days) [43]. In this study, decaying cow urine was effective in attracting gravid culicine spp from Day 4 to Day 30. The oviposition trap evaluation in rural Tanzania captured both gravid *An. gambiae *s.l. and *Cx. quinquefasciatus *in natural habitats [44]. The density of mosquitoes collected could have been higher if cow urine was incorporated in those devices. This would increase cues emanated and influence collection as trends showed that both species share habitats.

According to our experiments, cow urine was effective as an oviposition attractant for *An. gambiae *s.l. in the first four days while for *Cx. quinquefasciatus *attractivity increased from day four to thirty in the dry season. This is worthwhile information that could be used to lure-and-kill gravid mosquitoes and hatched larvae. Even though there is a growing interest in using bio-pesticides such as fungal spores for mosquito larvae control [45], the main focus biases to the control of adult mosquitoes [46,47]. Therefore, a need to incorporate oviposition chemical cues for gravid mosquitoes and apply it in larval habitat control arises.

Oviposition attractants, which are locally available, low cost and reliable, should be deployed in aggregating mosquitoes' larval habitats for use in the planning and management of effective control programmes for mosquitoes. The impact of larval control is significant in reducing adult mosquitoes and malaria cases [48-51].

The bacterial colonies associated with the decomposition of cow urine in intervals of days, should be isolated to understand the species identity and chemical cue products responsible.

## Conclusions

The findings of this study suggest that cow urine evidently influences choice of oviposition site, which vary between species, and aged urine induces markedly different responses to fresh urine.

## Competing interests

The authors declare that they have no competing interests.

## Authors' contributions

EJK conceived and designed experiments. AMM and BJM conducted all the experimental work. EJK and BJM did data analysis and interpretation. EJK wrote the manuscript. EAO, MN, FM, BJM and AMM edited the manuscript. All authors approved the final version for submission.
